# Targeting young drinkers online: the effectiveness of a web-based brief alcohol intervention in reducing heavy drinking among college students: study protocol of a two-arm parallel group randomized controlled trial

**DOI:** 10.1186/1471-2458-11-231

**Published:** 2011-04-14

**Authors:** Carmen V Voogt, Evelien AP Poelen, Marloes Kleinjan, Lex ACJ Lemmers, Rutger CME Engels

**Affiliations:** 1 Behavioural Science Institute, Radboud University Nijmegen, Nijmegen, P.O. Box 9104, 6500 HE Nijmegen, the Netherlands; 2 Trimbos Institute, Netherlands Institute of Mental Health and Addiction, Utrecht, the Netherlands

## Abstract

**Background:**

The prevalence of heavy drinking among college students and its associated health related consequences highlights an urgent need for alcohol prevention programs targeting 18 to 24 year olds. Nevertheless, current alcohol prevention programs in the Netherlands pay surprisingly little attention to the drinking patterns of this specific age group. The study described in this protocol will test the effectiveness of a web-based brief alcohol intervention that is aimed at reducing alcohol use among heavy drinking college students aged 18 to 24 years old.

**Methods/Design:**

The effectiveness of the What Do You Drink web-based brief alcohol intervention will be tested among 908 heavy drinking college students in a two-arm parallel group randomized controlled trial. Participants will be allocated at random to either the experimental (*N *= 454: web-based brief alcohol intervention) or control condition (*N *= 454: no intervention). The primary outcome measure will be the percentage of participants who drink within the normative limits of the Dutch National Health Council for low-risk drinking. These limits specify that, for heavy alcohol use, the mean consumption cannot exceed 14 or 21 glasses of standard alcohol units per week for females and males, respectively, while for binge drinking, the consumption cannot exceed five or more glasses of standard alcohol units on one drinking occasion at least once per week within one month and six months after the intervention. Reductions in mean weekly alcohol consumption and frequency of binge drinking are also primary outcome measures. Weekly Ecological Momentary Assessment will measure alcohol-related cognitions, that is, attitudes, self-efficacy, subjective norms and alcohol expectancies, which will be included as the secondary outcome measures.

**Discussion:**

This study protocol describes the two-arm parallel group randomized controlled trial developed to evaluate the effectiveness of a web-based brief alcohol intervention. We expect a reduction of mean weekly alcohol consumption and frequency of binge drinking in the experimental condition compared to the control condition as a direct result of the intervention. If the website is effective, it will be implemented in alcohol prevention initiatives, which will facilitate the implementation of the protocol.

**Trial registration:**

Netherlands Trial Register NTR2665.

## Background

The prevalence of heavy alcohol use among young adults and its associated health related consequences has become a great public health concern in most Western countries [1]. The percentage of heavy drinkers is particularly high among college students [2-5] and those who are affiliated with fraternities and sororities [6-8]. In the Netherlands, a substantial number of young adults engages in heavy alcohol use [3]. Heavy alcohol use can have detrimental short and long-term health related consequences for young adults, including risky sexual behaviour [9], brain damage [10], problematic alcohol use in adulthood [11], liver damage [12], and various types of cancer [1]. Heavy drinking among young adults and its social and economic burden highlights an urgent need to develop alcohol prevention programs targeted at 18 to 24 year olds. Nevertheless, current alcohol prevention programs in the Netherlands pay surprisingly little attention to young adults' drinking patterns. The study described in this protocol will test the effectiveness of a web-based brief alcohol intervention aimed at reducing alcohol use among heavy drinking college students aged 18 to 24 years old.

Previous studies have found that web-based brief alcohol interventions or individual single-session interventions without therapeutic guidance can be effective in reducing heavy alcohol use among young adults and students [13-19]. Originally, brief alcohol interventions were delivered using conventional methods, such as face-to-face [20,21] and postal mail methods [22]. Recently, interventions have been delivered electronically via computer programs [17] and Internet [16,23]. This web-based approach may have a number of advantages over the more traditional delivery methods. First, heavy drinkers are generally not interested in any type of treatment because they either do not think of themselves as heavy drinkers or they do not recognize that their drinking patterns may cause serious health risks; therefore, they use interventions without therapeutic involvement rather than group and individual counselling treatments to address their drinking behaviour [13]. Second, web-based brief alcohol interventions allow easy access to large audiences. Third, such interventions allow participants to access the intervention at their own convenience, which may enhance participants' feelings of privacy and anonymity. Fourth, these types of interventions are brief; therefore, less time-consuming and easier to implement. Finally, tailored information can be provided in an automated, cost-effective and flexible way [24]. Therefore, web-based brief alcohol interventions may be particularly suitable for our target population, especially considering that the majority of young adults in Western countries have access to the Internet and make frequent use of Internet technologies [25,26].

### Objectives and hypotheses

The objective of our study is to assess the effectiveness of the web-based brief alcohol intervention What Do You Drink (WDYD) among heavy drinking college students aged 18 to 24 years old. The effectiveness of the intervention will be tested at one month and six months after the intervention. In total, five pre-tests and 26 post-tests will be assessed weekly using Ecological Momentary Assessment (EMA) [27]. We expect that a larger percentage of participants in the intervention condition will drink within the normative limits of the Dutch National Health Council for low-risk drinking [28] compared to the control condition as a direct result of the intervention. This means that their consumption will not exceed a mean heavy alcohol use consumption of more than 14 or 21 glasses of standard alcohol units per week for females and males, respectively and/or, in case of binge drinking, five or more glasses of standard alcohol units on one drinking occasion at least once per week within one month and six months after the intervention. One standard alcohol unit contains ten grams of ethanol. Moreover, it is hypothesized that participants in both arms of the intervention would reduce their mean weekly alcohol consumption and frequency of binge drinking; although, it is expected that the exposure to the WDYD web-based brief alcohol intervention will be more effective compared to no intervention.

## Methods/Design

### Trial design

The effectiveness of the web-based brief alcohol intervention for heavy drinking college students will be tested in a two-arm parallel group randomized controlled trial. Participants will comprise 908 heavy drinking college students aged 18 to 24 years old. They will be randomly assigned to either the experimental (*N *= 454: web-based brief alcohol intervention) or control condition (*N *= 454: no intervention).

### Participants

A convenience sampling strategy will be used to recruit participants from Higher Professional Education (HBO) Institutions and Universities in the Netherlands. We will recruit participants by distributing flyers at the HBO Institutions and Universities and sending e-mails with information about the study to college students. Respondents will be given an e-mail address to obtain additional information about the study. Then, they will be invited to complete an online screening questionnaire to establish whether they fulfil the inclusion criteria. The online screening questionnaire contains items on demographic characteristics, alcohol use, and willingness to change drinking behaviour. To fulfil the inclusion criteria participants have to: 1) be between 18 and 24 years old, 2) report heavy drinking in the past six months, 3) be willing to change alcohol consumption, 4) have access to the Internet, and 5) sign an informed consent. Heavy drinking is defined based on the above-mentioned definition of heavy alcohol use and binge drinking, and it differs across participants' sex. Participants should be either heavy alcohol users and/or binge drinkers to fulfil the inclusion criteria. College students showing symptoms of alcohol abuse or dependence, that is an AUDIT score of 20 or above [29], and/or receiving treatment for alcohol-related problems, will be excluded from the sample. Participants satisfying the inclusion criteria will be invited by e-mail to electronically sign the informed consent containing information about confidentiality, voluntary participation, and human subject protections. Approval for the design and data collection was already obtained from the Ethical Committee (ECG) of the Faculty of Social Sciences of Radboud University Nijmegen in the Netherlands.

### Interventions

The web-based brief alcohol intervention, What Do You Drink (WDYD), aims to detect and reduce heavy drinking of young adults who are willing to decrease their alcohol consumption, preferably below the Dutch guidelines for low-risk drinking. The intervention is based on Motivational Interviewing [30] and parts of the I-Change model [31] and focuses predominantly on the action phase of the behaviour change process. Knowledge, social norms, and self-efficacy are embedded as the most changeable determinants of behaviour change (Voogt CV, Poelen EAP, Kleinjan M, Engels RCME: The development of a web-based brief alcohol intervention in reducing heavy drinking among college students: An Intervention Mapping approach, submitted).

The theoretical underpinning of web-based brief alcohol interventions is based on the literature on Motivational Interviewing [30] and social influence [32]. Motivational Interviewing, "a client-centred, directive method for enhancing intrinsic motivation to change by exploring and resolving ambivalence" [30], includes goal setting and action planning components [33]. A basic element in these types of interventions is the presentation of discrepant personal information to increase an individual's motivation to change or modify his or her behaviour [23]. A web-based brief alcohol intervention could present this discrepancy in two parts: a screening procedure and personalized feedback that is based on the screening outcomes. Topics that are addressed in the screening and the personalized feedback include personal drinking profile, risk factors, and normative comparisons. The inclusion of normative feedback is based on theory about social influence [32]. This type of feedback offers comparative information about personal drinking levels and drinking levels of a relevant comparison group, such as same-sex peers [23]. The use of personalized feedback implies that the intervention is "tailored" to the individuals' personal situation. Tailored interventions might be more effective than general interventions because the receiver of the intervention identifies him or herself with the personal-related information and pays more attention to the message and because they contain more relevant and less redundant information compared to general interventions [34].

The first part of WDYD focuses on the motivation phase of the behaviour change process and contains a homepage and a screening test with personalized feedback. The principle of a screening procedure with personalized feedback on alcohol-related knowledge and social norms has been shown effective when used in web-based brief alcohol interventions [15,16,19,35]. The screening test includes items addressing participants' name, sex, age, education level, weight, alcohol use, willingness to change alcohol consumption, average expenses on consumed alcohol beverages, and descriptive social norms. After completing the screening test, participants will receive personalized feedback that will depend on their answers to the questions on the screening test. The feedback will be tailored to participants' sex, alcohol intake, and perceived social norm. It will provide 1) advice about drinking according to the guidelines of the Dutch National Health Council, recommending that men should not drink more than two glasses alcohol per day and women one glass alcohol per day [28]. Further, it will provide information about 2) the amount of glasses of standard alcohol units that the participant consumed in the last year, with estimates of the number of calories consumed, the amount of weight added because of drinking, and the amount of money spent on drinking. Lastly, it will depict 3) a bar chart comparing the number of glasses of standard alcohol units per week that participants think their same-sex peers consume with the number of glasses of standard alcohol units per week that participants' same-sex peers actually consume. The comparative data of the descriptive social norms from a proximal reference group will be retrieved from alcohol prevalence estimates for the same-sex groups found in a nationally representative sample of the general population [36]. After receiving personalized feedback, participants will be offered access to the second part of the intervention via a registration and sign-up procedure.

The second part of WDYD focuses on the action phase of the behaviour change process, with a general goal of reducing heavy drinking. Specific proximal (short-term) goals, also called action plans, are found to be more effective compared to distal (long-term) goals [33]. Therefore, participants will be prompted to make decisions about the maximum amount of glasses of standard alcohol units they want to drink on every day of the week at a given point of time, preferably within the limits of low-risk drinking.

In addition to goal setting and action planning, the WDYD intervention will include self-efficacy. A substantial number of studies have indicated that adolescents with low self-efficacy for avoiding heavy drinking in social situations are more likely to engage in heavy drinking [37-39]. Therefore, WDYD focuses on strengthening participants' drinking refusal self-efficacy [40] by proving tips to resist alcohol in different drinking situations, which is expected to lead to behavioural change to succeed and maintain drinking goals. Participants will be asked to choose three out of the twelve provided drinking situations (derived from the Young's drinking refusal self-efficacy questionnaire (DRSEQ-RA: [38,41])). Subsequently, participants will be asked to give a rationale why they find it hard to resist alcohol in the three chosen drinking situations.

Finally, several tips will be offered for each of the chosen drinking situations to help participants cope with these situations in order to succeed and maintain general and specific drinking goals.

### Intervention conditions

Participants will be randomly assigned to either the experimental condition - exposure to the WDYD intervention - or the control condition - no intervention.

### Data collection

**Figure 1:**
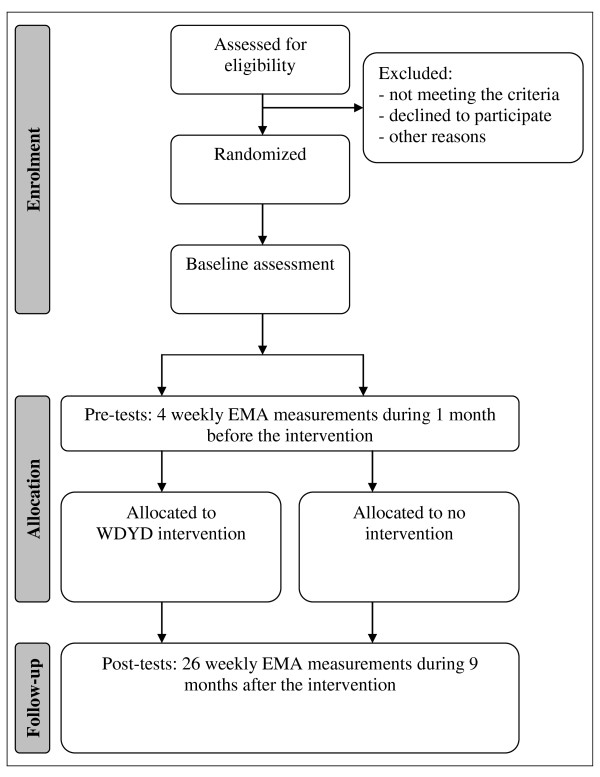
**Study design**.

An overview of measurements is given in Figure 1. The baseline assessment and pre-tests (one month before the intervention) are already collected in January 2011. The post-tests data will be obtained from weekly EMA measurements collected over 26 fixed time points following the intervention, that is, from February until August 2011, with a final follow-up in November 2011. Every Monday, participants will report 1) the number of glasses of standard alcohol units they drank during each day of that week, 2) the frequency of binge drinking within that week, and 3) the frequency of drinking and their ability to resist 26 different drinking situations during that week. Attitudes, self-efficacy, subjective norms and alcohol expectancies will be measured at baseline, immediately after the intervention, and one and six months after the intervention. In addition, a cost-effectiveness evaluation will be conducted along with the RCT, with follow-ups at baseline and one and six months. After completing the final follow-up, all participants will receive a monetary reward of hundred euro.

### Outcomes

The primary outcome measure will be the percentage of participants who drink within the normative limits of the Dutch National Health Council for low-risk drinking. Thereby, their mean consumption rate cannot exceed 14 or 21 glasses of standard alcohol units per week for females and males, respectively, and/or five or more glasses of standard alcohol units on one drinking occasion at least once per week within one month and six months after the intervention. In addition, reductions in mean weekly alcohol consumption and frequency of binge drinking will also be included as primary outcome measures.

Weekly alcohol consumption will be measured with the Dutch version of the Alcohol Weekly Recall [42]. Respondents will be asked to indicate retrospectively how many glasses of standard alcohol units they consumed in the last seven days. To ensure standardized responses, an overview of standard units for various beverages will be provided. The frequency of binge drinking will be measured by asking respondents how often they consumed five or more glasses of standard alcohol units on one drinking occasion at least once per week in the past week. They will be asked to respond on a 7-point scale ranging from (1) "never" to (7) "every day".

Weekly EMA will be employed in the pre-tests and post-tests to assess the secondary outcome measures. EMA is a generic term encompassing various research methods that utilize repeated measurements to assess people's current or very recent states or behaviours in their natural environments according to strategically selected moments in time [27]. One of the advantages of EMA is that it contains measures that are ecologically more valid, as data are collected in real-world environments. The most relevant advantage of EMA is that it reduces bias due to memory effects because it assesses the participants' most recent alcohol use instead of asking them to recall their past alcohol consumption. This enhances the validity of self-reports [43]. Scholars who have employed EMA using different designs showed that EMA is a useful methodology for assessing drinking patterns [44].

### Sample size

The sample size for our study will be based on a power calculation for detecting an increase in the percentage of participants showing low-risk drinking (i.e., who do not show heavy drinking) after one month of 42% in the experimental group versus 31% in the control group (Boon B, Risselada A, Huiberts A, Smit F: Reduced alcohol consumption in male adults due to a one time computer tailored advice: A randomised controlled trial, in press). When using a 2-sided test at alpha = 0.05, a power of (1-beta) = 0.80, and expecting a worst case scenario of 30% loss-to-follow-up after randomization, we will need a total sample size of 908 respondents (*N *= 454 per condition).

### Randomization

An independent researcher of the Behavioural Science Institute will randomly assign participants to the experimental and the control condition before baseline assessment. Randomization will be carried out centrally using a blocked randomization scheme (block size 4), and it will be stratified by sex and education level, as the Dutch guidelines for low-risk drinking differ for men and women.

### Statistical methods

To test the effectiveness of the web-based brief alcohol intervention, significantly more participants in the experimental condition would need to fulfil the criteria for low-risk drinking at one and six months follow-up compared to participants in the control condition; therefore, we will employ binomial statistical analyses to assess differences between the control and experimental conditions. Logistic regression models in SPSS and/or Mplus will be analyzed to test how the intervention relates to aggregated measures of drinking one month, three months, and six months after the intervention as well as at the final follow-up. The effect sizes as well as confidence intervals will be reported to determine both the magnitude and effect of the web-based brief alcohol intervention on heavy drinking. In addition, we will test whether age, sex, and drinking status moderate the main effect of the intervention on heavy drinking. We are also interested in possible mediators in the relation between the web-based brief alcohol intervention and alcohol consumption. Using SPSS and/or Mplus, we will test whether attitudes, social norms, and self-efficacy (ASE-model) could mediate the main effect because WDYD focuses predominantly on the latter two alcohol-related cognitions. The EMA data will comprise a large number of observations for each participant, making it possible to examine alcohol use of each participant over time. Therefore, in addition to the binomial statistical analyses, we will examine the effect of the intervention on trajectories and growth curves of alcohol consumption [45]. The EMA enables us to examine specific point in time at which the intervention is most successful and its effect size starts decreasing. Further, HLM survival analyses will be conducted to test the efficacy of the intervention. Moreover, a cost-effectiveness evaluation will be carried out.

## Discussion

The present study protocol presents two-arm parallel group randomized controlled trial evaluating the effectiveness of the WDYD intervention for 18 to 24 years old college students. WDYD aims to detect and reduce heavy drinking of young adults, preferably below the Dutch guidelines for low-risk drinking. It is hypothesized that reductions in mean weekly alcohol consumption and frequency of binge drinking will occur in both arms, but exposure to the WDYD web-based brief alcohol intervention will be more effective compared to no intervention.

### Strengths and limitations

The first strength of the WDYD intervention is that it incorporates elements of theory on Motivational Interviewing and social influence, which have been proven to be effective when used in web-based brief alcohol interventions aimed at reducing heavy drinking among students [13-17,19]. Second, WDYD is a tailored web-based brief alcohol intervention that may offer a more beneficial approach compared to the traditionally delivered interventions, especially for young adults [23]. Third, the use of EMA measurements in the study reduces recall bias, which enhances the validity of self-reports [43]. A limitation of the study is that the behaviour of young adults will be based entirely on self-report measures, which may be subject to over- or underreporting of alcohol use due to social desirability [46]. However, evidence suggests that self-report measures of alcohol use are reliable and valid when confidentially is assured [47,48]. In addition, participants will not be explicitly informed about the selection variables in order to avoid stigmatization. However, selecting participants and providing accurate study information to the participants is a general ethical issue with targeted interventions [49].

### Implications for practice

The insights that will be obtained from the WDYD effectiveness study will be communicated to scientists and health professionals. Moreover, if proven effective, the WDYD intervention will be further implemented in existing alcohol prevention initiatives. The collaboration with the Trimbos Institute (Netherlands Institute of Mental Health and Addiction) provides a high potential to ensure effective distribution of information and an adequate large-scale implementation, since WDYD can be easily incorporated in their materials and programs.

## Conclusion

This study has described a study protocol for testing an intervention aimed at reducing heavy drinking among college students. Evaluation of the intervention will provide insights into the effectiveness of WDYD and the precursors of alcohol use among college students aged 18 to 24 year olds.

## Competing interests

The authors declare that they have no competing interests.

## Authors' contributions

CV is responsible for the data collection and data analysis, as well as for reporting the study results. All others authors are supervisors and grant applicators. All authors read and approved the final protocol.

## Pre-publication history

The pre-publication history for this paper can be accessed here:

http://www.biomedcentral.com/1471-2458/11/231/prepub

## References

[B1] Rehm J Mathers C Popova S Thavorncharoensap M Teerawattananon Y Patra J Global burden of disease and injury and economic cost attributable to alcohol use and alcohol-use disorders The Lancet 2009 373 9682 2223 2233 10.1016/S0140-6736(09)60746-7 19560604

[B2] Dawson DA Grant BF Stinson FS Chou PS Another look at heavy episodic drinking and alcohol use disorders among college and noncollege youth Journal of Studies on Alcohol and Drugs 2004 65 4 477 488 10.15288/jsa.2004.65.477 15378804

[B3] Graaf Rd ten Have M van Dorsselaer S De psychische gezondheid van de Nederlandse bevolking 2010 Utrecht: Trimbos Instituut

[B4] Karam E Kypri K Salamoun M Alcohol use among college students: an international perspective Current Opinion in Psychiatry 2007 20 3 213 221 17415072 10.1097/YCO.0b013e3280fa836c

[B5] Kypri K Cronin M Wright CS Do university students drink more hazardously than their non-student peers? Addiction 2005 100 5 713 714 10.1111/j.1360-0443.2005.01116.x 15847629

[B6] Ham LS Hope DA College students and problematic drinking: A review of the literature Clin Psychol Rev 2003 23 5 719 759 10.1016/S0272-7358(03)00071-0 12971907

[B7] Maalsté N Ad Fundum! Een blik in de gevarieerde drinkcultuur van het Nederlandse studentenleven 2000 Utrecht: Centrum Verslavings Onderzoek (CVO)

[B8] Turrisi R Larimer ME Mallett KA Kilmer JR Ray AE Mastroleo NR Geisner IM Grossbard J Tollison S Lostutter TW Montoya H A randomized clinical trial evaluating a combined alcohol intervention for high-risk college students Journal of Studies on Alcohol and Drugs 2009 70 4 555 567 19515296 10.15288/jsad.2009.70.555 PMC2696296

[B9] Hingson RW Zha WX Weitzman ER Magnitude of and trends in alcohol-related mortality and morbidity among U.S. college students ages 18-24, 1998-2005 Journal of Studies on Alcohol and Drugs 2009 12 20 19538908 10.15288/jsads.2009.s16.12 PMC2701090

[B10] Zeigler DW Wang CC Yoast RA Dickinson BD McCaffree MA Robinowitz CB Sterling ML Assoc AM The neurocognitive effects of alcohol on adolescents and college students Prev Med 2005 40 1 23 32 10.1016/j.ypmed.2004.04.044 15530577

[B11] O'Neill SE Parra GR Sher KJ Clinical relevance of heavy drinking during the college years: Cross-sectional and prospective perspectives Psychology of Addictive Behaviors 2001 15 4 350 359 11767268 10.1037//0893-164x.15.4.350

[B12] Norstrom T Ramstedt M Mortality and population drinking: a review of the literature Drug and Alcohol Review 2005 24 6 537 547 10.1080/09595230500293845 16361210

[B13] Chiauzzi E Green TC Lord S Thum C Goldstein M My student body: A high-risk drinking prevention web site for college students J Am Coll Health 2005 53 6 263 274 10.3200/JACH.53.6.263-274 15900990 PMC1885481

[B14] Kypri K Saunders JB Williams SM McGee RO Langley JD Cashell-Smith ML Gallagher SJ Web-based screening and brief intervention for hazardous drinking: a double-blind randomized controlled trial Addiction 2004 99 11 1410 1417 10.1111/j.1360-0443.2004.00847.x 15500594

[B15] Doumas DM McKinley LL Book P Evaluation of two web-based alcohol interventions for mandated college students J Subst Abuse Treat 2009 36 1 65 74 10.1016/j.jsat.2008.05.009 18657941

[B16] Kypri K Hallett J Howat P McManus A Maycock B Bowe S Horton NJ Randomized controlled trial of proactive web-based alcohol screening and brief intervention for university students Arch Intern Med 2009 169 16 1508 1514 10.1001/archinternmed.2009.249 19752409

[B17] Neighbors C Larimer ME Lewis MA Targeting misperceptions of descriptive drinking norms: Efficacy of a computer-delivered personalized normative feedback intervention J Consult Clin Psychol 2004 72 3 434 447 10.1037/0022-006X.72.3.434 15279527

[B18] Neighbors C Lewis MA Being controlled by normative influences: Self-determination as a moderator of a normative feedback alcohol intervention Health Psychol 2006 25 5 571 579 10.1037/0278-6133.25.5.571 17014274 PMC2474672

[B19] Bewick BM Trusler K Barkham M Hill AJ Cahill J Mulhern B The effectiveness of web-based interventions designed to decrease alcohol consumption - A systematic review Prev Med 2008 47 1 17 26 10.1016/j.ypmed.2008.01.005 18302970

[B20] Borsari B Carey KB Effects of brief motivational intervention with college student drinkers Journal of Consultancy and Clinical Psychology 2000 68 28 33 10965648

[B21] Moyer A Finney JW Swearingen CE Vergun P Brief interventions for alcohol problems: a meta-analytic review of controlled investigations in treatment-seeking and non-treatment-seeking populations Addiction 2002 97 3 279 292 10.1046/j.1360-0443.2002.00018.x 11964101

[B22] Wild TC Cunningham JA Roberts AB Controlled study of brief personalized assessment-feedback for drinkers interested in self-help Addiction 2007 102 2 241 250 10.1111/j.1360-0443.2006.01682.x 17222278

[B23] Spijkerman R Roek MAE Vermulst A Lemmers L Huiberts A Engels RCME Effectiveness of a web-based brief alcohol intervention and added value of normative feedback in reducing underage drinking: A randomized controlled trial Journal of Medical Internet Research 2010 12 5 e65 10.2196/jmir.1465 21169172 PMC3057308

[B24] Riper H van Straten A Keuken M Smit F Schippers G Cuijpers P Curbing problem drinking with personalized-feedback interventions: a meta-analysis Am J Prev Med 2009 36 3 247 255 10.1016/j.amepre.2008.10.016 19215850

[B25] Gross EF Adolescent Internet use: What we expect, what teens report Journal of Applied Developmental Psychology 2004 25 6 633 649 10.1016/j.appdev.2004.09.005

[B26] The UCLA Internet Report. Surveying the digital future. Year three http://www.digitalcenter.org/pdf/InternetReportYearThree.pdf

[B27] Stone AA Shiffman S Ecological Momentary Assessment in behavioural medicine Ann Behav Med 1994 16 199-202

[B28] Gezondheidsraad Richtlijnen voor gezonde voeding 2006 [Guidlines for healthy nutrition 2006] 2006 Den Haag: Gezondheidsraad [Dutch National Health Council]

[B29] Babor T Higgins-Biddle JC Saunders J Monteiro MG The Alcohol Use Disorders Identification Test: Guidelines for use in primary care 2001 World Health Organization. Department of Mental Health and Substance Dependence 1 40

[B30] Miller WR Rollnick S Motivational interviewing: Preparing people for change 2002 New York: Guilford Press

[B31] De Vries H Dijkstra M Kuhlman P Self-efficacy: The third factor besides attitude and subjective norm as a predictor of behavioral intentions Health Educ Res 1988 3 273 282 10.1093/her/3.3.273

[B32] Bandura A Social foundations of thought and action: A social cognitive theory 1986 Englewood Cliffs, New Jersey: Prentice-Hall

[B33] Bodenheimer T Handley MA Goal-setting for behavior change in primary care: An exploration and status report Patient Educ Couns 2009 76 174 180 10.1016/j.pec.2009.06.001 19560895

[B34] De Vries H Brug J Computer-tailored interventions motivating people to adopt health promoting behaviors: Introduction to a new approach Patient Educ Couns 1999 36 99 105 10.1016/S0738-3991(98)00127-X 10223015

[B35] Walters ST Vader AM Harris TR A controlled trial of web-based feedback for heavy drinking college students Prevention Science 2007 8 1 83 88 10.1007/s11121-006-0059-9 17136461

[B36] Gezondheid, leefstijl, gebruik van zorg [Health, lifestyle, care use] http://statline.cbs.nl/StatWeb/publication/?DM=SLNL&PA=03799&D1=210-214,297&D2=0-17&D3=0&D4=a&VW=T

[B37] Lee NK Oei TPS Greeley JD The interaction of alcohol expectancies and drinking refusal self-efficacy in high and low risk drinkers Addict Res 1999 7 2 91 102 10.3109/16066359909004377

[B38] Oei TPS Hasking PA Young RM Drinking refusal self-efficacy questionnaire-revised (DRSEQ-R): a new factor structure with confirmatory factor analysis Drug Alcohol Depen 2005 78 3 297 307 10.1016/j.drugalcdep.2004.11.010 15893161

[B39] Young RM Connor JP Ricciardelli LA Saunders JB The role of alcohol expectancy and drinking refusal self-efficacy beliefs in university student drinking Alcohol Alcoholism 2006 41 1 70 75 10.1093/alcalc/agh237 16299109

[B40] Oei TPS Morawska A A cognitive model of binge drinking: The influence of alcohol expectancies and drinking refusal self-efficacy Addict Behav 2004 29 1 159 179 10.1016/S0306-4603(03)00076-5 14667427

[B41] Young RM Hasking PA Oei TPS Loveday W Validation of the drinking refusal self-efficacy questionnaire - Revised in an adolescent sample (DRSEQ-RA) Addict Behav 2007 32 4 862 868 10.1016/j.addbeh.2006.07.001 16919885

[B42] Lemmens P Tan ES Knibbe RA Measuring quantity and frequency of drinking in a general population survey: a comparison of five indices J Stud Alcohol 1992 53 5 476 486 1405641 10.15288/jsa.1992.53.476

[B43] Shiffman S Stone AA Hufford MR Ecological momentary assessment Annual Review of Clinical Psychology 2008 4 1 32 10.1146/annurev.clinpsy.3.022806.091415 18509902

[B44] Collins RL Morsheimer ET Shiffman S Paty JA Gnys M Papadonatos GD Ecological momentary assessment in a behavioural drinking moderation training program Experimental and Clinical Psychopharmacology 1998 6 306 315 10.1037/1064-1297.6.3.306 9725114

[B45] Van Zundert RM Ferguson SG Shiffman S Engels RCME Dynamic effects of self-efficacy on smoking lapses and relapse among adolescents Health Psychol 2010 29 3 246 254 10.1037/a0018812 20496978

[B46] Offer D Kaiz M Howard KI Bennett ES The altering of reported experiences J Am Acad Child Psy 2000 39 6 735 742 10.1097/00004583-200006000-00012 10846308

[B47] Engels RCME Van der Vorst H Dekovic M Meeus W Correspondence in collateral and self-reports on alcohol consumption: A within family analysis Addict Behav 2007 32 5 1016 1030 10.1016/j.addbeh.2006.07.006 16952426

[B48] Winters KC Stinchfield RD Henly GA Schwartz RH Validity of adolescent self-report of alcohol and other drug involvement Int J Addict 1991 25 11A 1379 1395 10.3109/10826089009068469 2132719

[B49] Lammers J Goossens F Lokman S Monshouwer K Lemmers L Conrod P R W Engels RCME Kleinjan M Evaluating a selective prevention programme for binge drinking among young adolescents: study protocol of a randomized controlled trial BMC Public Health 2011 11 126 21338506 10.1186/1471-2458-11-126 PMC3053243

